# Obsessive-compulsive symptoms and aggression in an adolescent with left temporal lobe agenesis: a case report

**DOI:** 10.1192/j.eurpsy.2026.11136

**Published:** 2026-07-31

**Authors:** S. Z.-Y. Ngui, R. H. Y. Loy

**Affiliations:** Department of Developmental Psychiatry, Institute of Mental Health, Singapore, Singapore

## Abstract

**Introduction:**

Temporal lobe agenesis is a rare neurodevelopmental anomaly. Psychiatric manifestations from temporal lobe pathology is well-reported in literature, however most evidence links to abnormalities such as temporal lobe epilepsy, lesions, hypoplasia or surgical resections (Vinte et al., 2021). The manifestation of aggressive and obsessive-compulsive symptoms in an adolescent with temporal lobe agenesis may offer insight into the neural underpinnings of social and emotional regulation pathways in the temporal lobe.

We report a 16-year-old boy with temporal lobe agenesis who presented with worsening aggression, obsessive questioning, compulsive urges, and persistent mental checking routines that first manifested at 13-years of age. Other symptoms of note include the urge to make inappropriate sexualized comments toward strangers, excessive concern over the legality of trivial decisions or actions, intense dislike of his family’s religion, and spending excessive time toileting or showering. Speech was also noted to be excessively formal.

**Objectives:**

To illustrate the psychiatric manifestations in a case of left temporal lobe agenesis, and treatment options utilized in this case.

**Methods:**

Clinical and corroborative histories were obtained, including patient, parents and school. Behaviours were observed for a period in a psychiatric ward. Informed consent was obtained and information was de-identified to ensure anonymity. The patient remains on ongoing follow-up.

Neuroimaging was done after he presented to a restructured hospital, as part of workup for altered mental state. He has no family history of psychiatric disorders. No epileptiform activity was observed since young. Developmental history noted speech delay and repetitive crawling behaviours, with no other significant concerns. The individual had previously demonstrated good academic performance, performing well in his primary school and secondary school exams.

**Results:**

A combination of ongoing behavioural therapy, psychoeducation, and psychotropic management was provided. Partial improvement in obsessive behaviour and reduction in aggressive episodes was observed.

**Conclusions:**

The link between psychiatric phenomology and temporal lobe abnormalities are well-documented. This case report only describes the coexistence of OCD/aggression in a patient with temporal lobe agenesis, and does not indicate a causal relationship between the two. However, more data may potentially suggest a trend over time. An existing case report notes similar obsessive-compulsive symptoms in a male paediatric patient with temporal lobe porencephaly (Deng et al., 2025). Our case highlights the potential for temporal lobe agenesis to also contribute to complex psychiatric manifestations. Such a pattern emphasizes the importance of comprehensive neuropsychiatric evaluation and symptom-focused treatment in atypical adolescent behavioural presentations.

**Disclosure of Interest:**

None Declared

